# Bronchial wall necrosis secondary to mucormycosis following SARS-Cov2 infection: A case report^☆^

**DOI:** 10.1016/j.radcr.2022.05.049

**Published:** 2022-06-17

**Authors:** Ghazal Arjmand, Elham Askari, Arya Kazemi, Ehsan Zarei, Sara Haseli, Nazanin Sadraei

**Affiliations:** a Medical student at Shahid Beheshti University of Medical Sciences, Tehran, Iran; b Chronic Respiratory Diseases Research Center, National Research Institute of Tuberculosis and Lung Diseases (NRITLD), Shahid Beheshti University of Medical Sciences, Tehran, Iran; c Department of Radiology, Shohada-E-Tajrish Hospital, Shahid Beheshti University of Medical Sciences, Tehran, Iran; d Medical Imaging Research Center, Shiraz University of Medical Sciences, Shiraz, Iran

**Keywords:** COVID-19, Diabetes, Mucormycosis, Chest CT, Bronchial necrosis, Lung collapse

## Abstract

Coronavirus 2019 infection (COVID-19) has a broad spectrum of clinical complications, some unrecognized. Herein, a case of a diabetic patient with multiple episodes of hemoptysis 2 months following her recovery from SARS-CoV-2 infection is reported. The initial computed tomography (CT scan) revealed the left lower lobe collapsed secondary to bronchial narrowing and obliteration. Bronchoscopy was performed, indicating necrotic endobronchial tissue, which was confirmed histopathologically as invasive mucormycosis. Bronchial necrosis due to mucormycosis is an unusual presentation of COVID-19-associated pulmonary mucormycosis. The accurate diagnosis could be challenging as it can resemble other pathologies such as malignancies. Therefore, it is crucial to identify this fatal complication in patients with prolonged COVID-19 and lung collapse.

## Introduction

Due to the recent burden of coronavirus 2019 infection (COVID-19), the detrimental manifestation of the acute respiratory syndrome of coronavirus 2 (SARS-CoV-2), such as superimposed mucormycosis, is being highlighted [1]. The widespread use of glucocorticoids and immunosuppressive therapy in COVID-19 patients to reduce the mortality rate puts the patients at risk of bacterial and fungal coinfections [2]. Mucormycosis caused by Mucorales species is a rare invasive opportunistic fungal infection that spreads rapidly in the hosts with predisposing factors such as hematologic malignancies, diabetes mellitus, solid organ transplantation, stem cell transplantation, neutropenia, prolonged corticosteroid use, and other immunocompromised situations [3]. Mucormycosis can be categorized into the following 6 categories based on the specific anatomical site of involvement: rhinocerebral, pulmonary, cutaneous, gastrointestinal, disseminated, and miscellaneous [4]. Pulmonary mucormycosis is the second most common presentation, with a high overall mortality rate [5]. Its symptoms are nonspecific, including dyspnea, cough, fever, hemoptysis, loss of appetite, weight loss, and night sweats. Significant bronchial necrosis and angioinvasion can be seen in severe cases [6]. In this article, a case report of a fatal pulmonary mucormycosis in a 77-year-old diabetic patient who presented with bronchial necrosis following her COVID-19 recovery is implicated.

## Case presentation

In November 2021, a 77-year-old female, a known case of hypertension and diabetes mellitus, was referred to our hospital-Masih Daneshvari hospital (Tehran, Iran) with recurrent nonmassive hemoptysis in the last 2 months. On admission, initial vital signs demonstrated a temperature of 36.5°C, heart rate of 100 bpm, respiratory rate of 19/min, oxygen saturation of 93%, and blood pressure of 150/100 mmHg. Decreased breath sound on the left side following dull precaution was detected on chest examination; other physical examinations were unremarkable. She had a history of hospital admission and a weight loss of 10 kg following her COVID-19 exposure 4 months prior. The patient has taken 2 doses of Sinopharm vaccine—manufactured in Beijing Institute of Biological Products—which the second dose was 1 month prior to her previous admission. During her previous admission due to COVID-19 pneumonia, she had received systemic corticosteroid, remdesivir, oral antibiotics (levofloxacin), and prophylactic anticoagulant (enoxaparin).

**Fig. 1:**
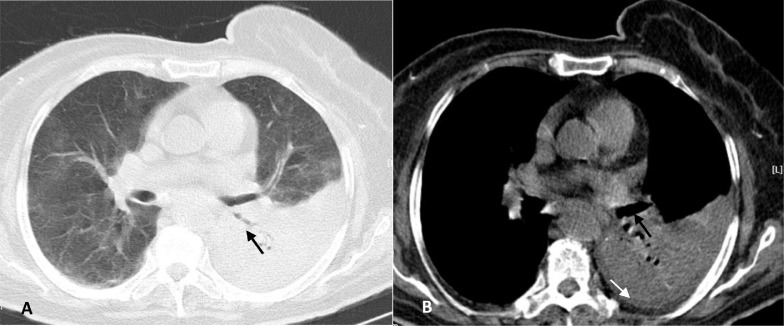
Noncontrast CT scan of lung (A: lung window, B: mediastinal window) shows narrowing and obliteration of the LLL bronchus (marked with black arrow) which resulted in LLL collapse/consolidation. Subtle left side pleural effusion is also noted (marked with white arrow).

**Fig. 2:**
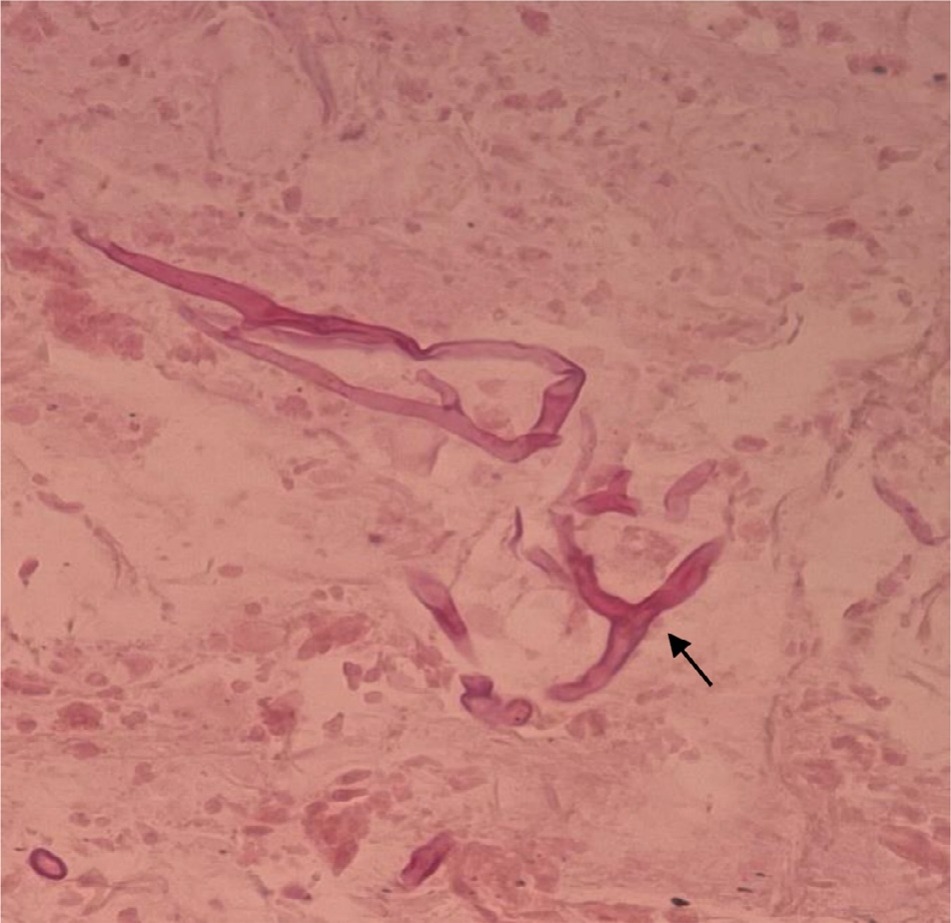
H and E stained sections reveals bronchial wall necrosis associated with broad pauciseptated hyaline hyphae (black arrow).

The laboratory test results on admission were as follows: random plasma glucose level = 302 mg/dl, hemoglobin A1c = 7.3%, leukocytes = 18.59 × 10^3^ /µl (neutrophils = 85%), platelet count = 534 × 10^3^ /µl, hemoglobin = 9.5 g/dl, C-reactive protein = 39.8 mg/l, erythrocyte sedimentation rate = 80 mm/h and lactate dehydrogenase = 617 U/l. Renal and liver function tests and venous blood gas values, are within normal range. The computed tomography (CT) scan of the chest on admission revealed left lower lobe (LLL) bronchus narrowing and obliteration, which had resulted in the LLL collapse with adjacent pleural thickening and effusion (Fig. 1). Bronchoalveolar lavage showed necrotic tissue, and the cytology results were negative regarding malignancy. Further histopathological examination of bronchial tissue showed broad, ribbon-like, pauciseptate hyphae with right-angle branching (Fig. 2). Antifungal treatment (Amphotericin) was initiated for the patient, and she underwent emergency angioembolization. The patient's hemoptysis continued despite the pulmonary embolization; therefore, urgent thoracotomy and left lower lobectomy was performed. Unfortunately, a few hours postoperation, the patient passed away.

## Discussion

The severity of COVID-19 disease varies from mild to severe, requiring medical treatments, including corticosteroids that may predispose the patients to invasive fungal infections such as mucormycosis [7], [8], [9].

Mucormycosis is a relatively rare infection caused by a group of fungi called Mucoromycetes, which has been increasing recently [6,10]. The most common species that can cause mucormycosis are Rhizopus, Mucor, and Rhizomucor. They can cause the disease in more than 70% of the patients [11]. They can rapidly invade different organs such as paranasal sinuses, lungs, kidneys, skin, gastrointestinal tract, and central nervous system. Generally, the clinical manifestation depends on the underlying comorbidity and the source of entry of the mucormycosis [12], [13], [14], [15]. Immunocompromised patients are the leading group who are affected by these microorganisms. In addition, infections caused by Mucorales are gradually being documented in patients with diabetes mellitus, neutropenia, chemotherapy, and steroid use [16], [17], [18], [19].Yet, the most rampant predisposing factor for mucormycosis in COVID-19 patients is diabetes mellitus [20], [21], [22]. The most prevalent clinical manifestations of mucormycosis are rhino-orbital-cerebral, pulmonary, cutaneous, gastrointestinal, and disseminated forms [3,23]. The mortality rate of pulmonary mucormycosis is high, estimated to be between 50% and 80%. Symptoms of mucormycosis depend on whereabouts of the location of the fungi development. The clinical manifestations of the disease are variable. Pulmonary mucormycosis symptoms could be nonspecific, including dyspnea, cough, fever, hemoptysis, and chest pain [24,25]. Therefore, the diagnosis of mucormycosis requires multilayered management that includes clinical symptoms, radiographic patterns, and fungal culture analysis [26]. Radiological findings in mucormycosis are diverse as they can be presented by solitary nodules and masses, lobar consolidations, cavitary lesions, and nonspecific infiltrations. However, the Halo and air crescent signs are less common [6,21,27,28]. Even though CT findings are nonspecific, the reversed halo sign can also be seen in pneumonia caused by aspergillosis [29]. Tracheobronchial mucormycosis is less common and accounts for less than half of all pulmonary mucormycosis cases. The major airways can become obstructed, and pulmonary blood vessels can be compromised by the endobronchial lesions, leading to a massive hemoptysis. This manifestation is more common in diabetic patients than those with malignancy [11,21,22]. The most common area to become involved is lobar bronchi dissemination to the upper lobes. The main-stem bronchi are the second most common parts involved, with no preference to the right or left side [6,21]. Usually, in addition to other findings, bronchoscopy could reveal mucosal necrosis, hyperemic mucosa, mass-like lesions, and purulent exudates [11]. The diagnosis highly depends on the histopathological analysis and fungal culture, whereas tissue swabs, bronchoalveolar lavage, and blood cultures are mostly nondiagnostic [30]. Fungal culture could assist the physician in identifying different species; however, it can also become false-negative in up to 50% of cases [31,32]. Histopathologically, the lesion is observed as a 5-15 μm diameter nonseptate or minimally separated hyphae. Compared to Aspergillosis, mucormycosis has ribbon-like hyphae with branching irregularity at the right angle under the microscope [30,32].

Hemoptysis can be caused mainly through invasion of the bronchial arterial circulation or, to a lesser degree, by nonbronchial and pulmonary arteries [33]. Hemoptysis is an infrequent finding in COVID-19 patients caused by anticoagulation therapy versus pulmonary embolism [34]. On the other hand, another infrequent factor leads to hemoptysis either during or after COVID-19 diagnosis, which is considered fatal. In this article, in an immunocompromised patient presented with hemoptysis post-COVID-19 pneumonia, mucormycosis was diagnosed. Early diagnosis and appropriate management could prevent further mortality.

This disease should be treated with combination therapy which includes the application of antifungal drugs and surgery. Studies have also indicated the effect of liposomal amphotericin B as an essential antifungal agent in managing mucormycosis. Isavuconazole or Posaconazole can also be used as adjuvant therapy. The prognosis of the therapy depends on many factors, such as early diagnosis and management to prevention of the spread of infection into the adjacent areas [8,31,35]. The surgical procedures that are recommended in the management of mucormycosis are lobectomy, pneumonectomy, or wedge resection, due to their ability to decrease the mortality rate and recurrence prevention [32,36].
